# The Homeopathic Revolution: Why Famous People and Cultural Heroes Choose Homeopathy

**DOI:** 10.1093/ecam/nen024

**Published:** 2011-06-05

**Authors:** Ellen Feingold

**Affiliations:** Director of the Homeopathy Center of Delaware, Wilmington, DE 19803, USA

Dana Ullman (ed.). *The Homeopathic Revolution*. North Atlantic Books, Berkeley, CA, 2007, page 386.

This is no ordinary book on homeopathy. The stunning scope of topics, the depth and breadth of knowledge and the abundant historical references throughout make this book a treasure. You will not want to miss a single word. (Caution: Do not skip Dana Ullman's *Notes*. All 143 *Notes* add flavor and zest to the story of homeopathy).

First, take a look at the *Table of Contents*, organized around occupations. It is a tour, hold onto your seats, around Dana Ullman's Renaissance brain.

Second, zip around the *Index* and try to think of anyone throughout the history of the past 200 years worth knowing about who did NOT seek or administer homeopathic medical care. Dana Ullman has included several hundred famous men and women with some connection to homeopathy, with so many testimonials of so many people who owe/owed their lives to homeopathy.

Even Moses, Dana Ullman tells us, was a would-be homeopath. Remember the Golden Calf incident? Moses ascended Mt Sinai to talk with God but his return to the people encamped at the foot of the mountain was delayed for 40 days. The people despaired. They became hopeless. They fashioned a Golden Calf and prayed to it for deliverance. Upon returning from the mountain with the Ten Commandments, Moses in great anger at the idolatry of his people smashed the Golden Calf. Then he pulverized it into powder, added it to water, and commanded the Israelites to drink of the solution. Moses did not know that he was using Aurum metallicum (gold) to treat despair and hopelessness, just as we homeopaths do today, thousands of years after Moses.

Third, take a look at the extensive *References* after every chapter. It is another glimpse into the far reaches of the author's intellect. The *References* range from Darwin to Medscape, from Jackson Pollack to Consumer Reports.

Now, delve into the book and let it carry you away. You will find fascinating stories of persons prescribing, using, or writing about homeopathy, most of it favorable, some not.

Over and over again we read stories of homeopathy getting a bad rap. The bad rap largely continues today, about 150 years after it started among Western-trained physicians. Dana helps us to understand the forces at work that resulted in the near destruction of a system of medicine as efficacious, gentle and healing as homeopathy.

Many of the book's testimonials in favor of homeopathy are in the form of personal letters written by one or another famous person to the author. (See, e.g., Sir Yehudi Menuhin's letter, page 161).

Do not miss the story of how the Flexner Report on Medical Education in 1910 all but destroyed homeopathy in America. Due to the Flexner Report all but two of the 22 homeopathic medical schools in 1900 had closed by 1923. Interestingly, the Flexner Report lauded the Johns Hopkins Medical School as the paragon of virtue. Its president at the time was, ironically, Ira Remsen, M. D., a graduate of the New York Homeopathic Medical College.

My only criticism of the book was that the fascinating glimpses of history through the prism of homeopathy were in some cases, all too brief. It left me wanting more detail.

Now, if only nonfamous people, like most of us, would choose homeopathy.

*Ellen Feingold* *Ellen Feingold*

